# The association of patient‐reported social determinants of health and hospitalization rate: A scoping review

**DOI:** 10.1002/hsr2.1124

**Published:** 2023-02-22

**Authors:** Ali Ardekani, Reza Fereidooni, Seyed Taghi Heydari, Sulmaz Ghahramani, Saeed Shahabi, Kamran Bagheri Lankarani

**Affiliations:** ^1^ Health Policy Research Center, Institute of Health Shiraz University of Medical Sciences Shiraz Iran

**Keywords:** healthcare utilization, hospital, inpatients, scoping review, social determinants of health

## Abstract

**Introduction:**

The interplay between social determinants of health (SDOH) and hospitalization is significant as targeted interventions can improve the social status of the individuals. This interrelation has been historically overlooked in health care. In the present study, we reviewed studies in which the association between patient‐reported social risks and hospitalization rate was assessed.

**Method:**

We performed a scoping literature review of articles published until September 1, 2022 without time limit. We searched PubMed, Embase, Web of Science, Scopus, and Google Scholar to find relevant studies using terms representing “social determinants of health” and “hospitalization.” Forward and backward reference checking was done for the included studies. All studies that used patient‐reported data as a proxy of social risks to determine the association between social risks and hospitalization rates were included. The screening and data extraction processes were done independently by two authors. In case of disagreement, senior authors were consulted.

**Results:**

Our search process retrieved a total of 14,852 records. After the duplicate removal and screening process, eight studies met the eligibility criteria, all of which were published from 2020 to 2022. The sample size of the studies ranged from 226 to 56,155 participants. All eight studies investigated the impact of food security on hospitalization, and six investigated economic status. In three studies, latent class analysis was applied to divide participants based on their social risks. Seven studies found a statistically significant association between social risks and hospitalization rates.

**Conclusion:**

Individuals with social risk factors are more susceptible to hospitalization. There is a need for a paradigm shift to meet these needs and reduce the number of preventable hospitalizations.

## 1 INTRODUCTION

Hospitalization is one of the most expensive aspects of healthcare service, accounting for one‐third of the total healthcare expenditure in the United States.^1^, ^2^, ^3^ Many health and insurance organizations have attempted to minimize the hospitalization rate by implementing preventive policies,^4^ with various clinical and epidemiological factors affecting this index.^5^, ^6^, ^7^ In recent years, several studies have demonstrated the substantial impact of social determinants of health (SDOH) on hospitalization rates.^8^, ^9^, ^10^, ^11^, ^12^, ^13^, ^14^, ^15^

The World Health Organization (WHO)^16^ defines the SDOH as “the conditions in which people are born, grown, work, live, and age, and the wider set of forces and systems shaping the conditions of daily life.” These conditions are influenced by a broader set of factors, including living conditions, economy, social policies, politics, and commercial determinants of health. SDOH can be a risk or protective factor based on their presence/absence or positive/negative valence. The term “social risks” is used to describe adverse individual‐level SDOH, such as food insecurity, unemployment, and housing instability.^17^ Addressing SDOH not only helps prevent the occurrence of diseases but also promotes public health and social equity.^18^

Although previous research has shown the relationship between healthcare utilization and social risks,^19^ it has often been general and lacked details. For example, many studies merely examined the relationship between age, sex, and the insurance status of individuals with healthcare utilization.^20^, ^21^, ^22^ It is also worth noting that the social construction of these characteristics, like the privileges and discriminations based on ageism, sexism, etc., has a much larger role in determining risk or protection than the characteristics themselves. The outlined approaches often lack a comprehensive evaluation of other social risks like food insecurity, neighborhood status, educational background, social isolation, and economic stability, which can provide a more accurate understanding of the social components that affect individuals' health. While general demographic characteristics (age, gender, etc.) are worthwhile, they can lead to nonspecific and often less valuable findings for policymaking. Given the interplay and integrity of SDOH dimensions, evaluating their impact as a system will provide a more accurate assessment of their effect on hospitalization.^13^

As the association between SDOH and hospitalization rate has gained attention recently and the potential size and scope of available literature and nature and extent of evidence were yet to be established, we used a scoping review design to elucidate the possible association between patient‐reported SDOH and hospitalization rate.

## 2 METHODS

The current scoping review followed the Joanna Briggs Institute (JBI) guidelines for scoping reviews^23^, ^24^ (Table 1) and the Preferred Reporting Items for Systematic Reviews and Meta‐Analyses Extension for Scoping Reviews (PRISMA‐ScR).^25^ The Institutional Review Board of Shiraz University of Medical Sciences assessed and approved the protocol of this study.

**Table 1:** Methodological steps.

Step	Topic	Description
1	Determination of the study question and objective	What is the impact of SDOH on the hospitalization rate?
2	Determination of the inclusion criteria	Participants: individuals who filled out a questionnaire on SDOH (at least three domains according to the thematic analysis). Concept: the relationship between patient‐reported SDOH and hospitalization. Context: community or hospital.
3	Description of the search strategy	Relevant keywords and MeSH terms were used (see Table S1).
4	Evidence‐based search	PubMed, Scopus, Web of Science, Embase, and Google Scholar were searched.
5	Evidence selection	Two independent authors screened the titles, abstracts, and full texts. Consultation with most expert authors put forth in case of disagreements.
6	Data extraction	Authors' names, publication year, study design and method, results, and conclusion were extracted.
7	Charting	Displaying the characteristics of the included studies.
8	Summarizing data	Determining the main themes and sub‐themes from the included studies using thematic analysis.
9	Consultation with experts	Two external experts in medical sociology and public health reviewed the framework.

Abbreviation: SDOH, Social Determinants of Health.

### 2.1 Search strategy

The electronic PubMed, Embase, Web of Science, and Scopus databases were searched until May 1, 2022, using relevant keywords and MeSH terms representing “social determinants of health” and “hospitalization” in the titles and abstracts. An update search was also done on September 1, 2022. We performed forward and backward reference checking for the included studies to identify any other relevant articles. Furthermore, Google Scholar was searched to retrieve gray literature, and relevant journals were manually searched for relevant studies (Table S1).

### 2.2 Screening process

The initial citations were imported into Endnote X9 for screening, and duplicates were removed. The title and abstract of each study were reviewed separately by two authors (AA and RF). Then, the full texts were assessed for eligibility. The screeners were blinded to each other's decisions. During the first and second screening processes, all conflicts in decisions were resolved by consultation with a third author (KBL or STH).

### 2.3 Eligibility criteria

For the inclusion of the studies, the participants, concept, and context were primarily defined (Table 1). We included studies in which (a) individuals reported their social needs (at least three aspects according to the framework described below), (b) the authors used quantitative investigation of the relationship between patient‐reported SDOH and hospitalization rate, and (c) the design of the study was community‐based or hospital‐based. We excluded studies in which (a) merely readmissions (planned or unplanned) were investigated, (b) studies that did not ask about social needs and used predefined data (like chart reviews) as a stand‐in rather than patient‐reported data, and (c) conference reports, review articles, and commentaries. There was no exclusion owing to the age of the participants, sample size, date, language, or publication country.

### 2.4 Data extraction, quality appraisal, and analysis

Using an Excel spreadsheet template, the data from the included studies were retrieved by two independent authors (AA and SS). The first author's surname, publication year, the year it was carried out, the purpose of the study, results, and conclusion were extracted. Also, the quality of the studies was measured independently using the Joanna Briggs Institute (JBI) checklist according to the study designs.^26^ The extraction of the data for two of the included studies was piloted to test spreadsheet usability before the main data extraction. Disagreements were solved by consensus with the author with the most expertise in the topic (KBL and SG).

We used thematic analysis to ascertain aspects of SDOH assessed in each study.^27^ First, we reviewed the extracted articles and categorized the aspects of SDOH in each study as initial codes. Then, we tried to fit the initial codes into the main themes of the Kaiser Family Foundation Framework (KFF)^28^ for SDOH. In addition to KFF, two new themes emerged. Two external experts reviewed the final themes in addition to the authors. The relationship between each domain of SDOH, including odds ratios or prevalence of social risks, was also extracted.

## 3 RESULTS

**Figure 1:**
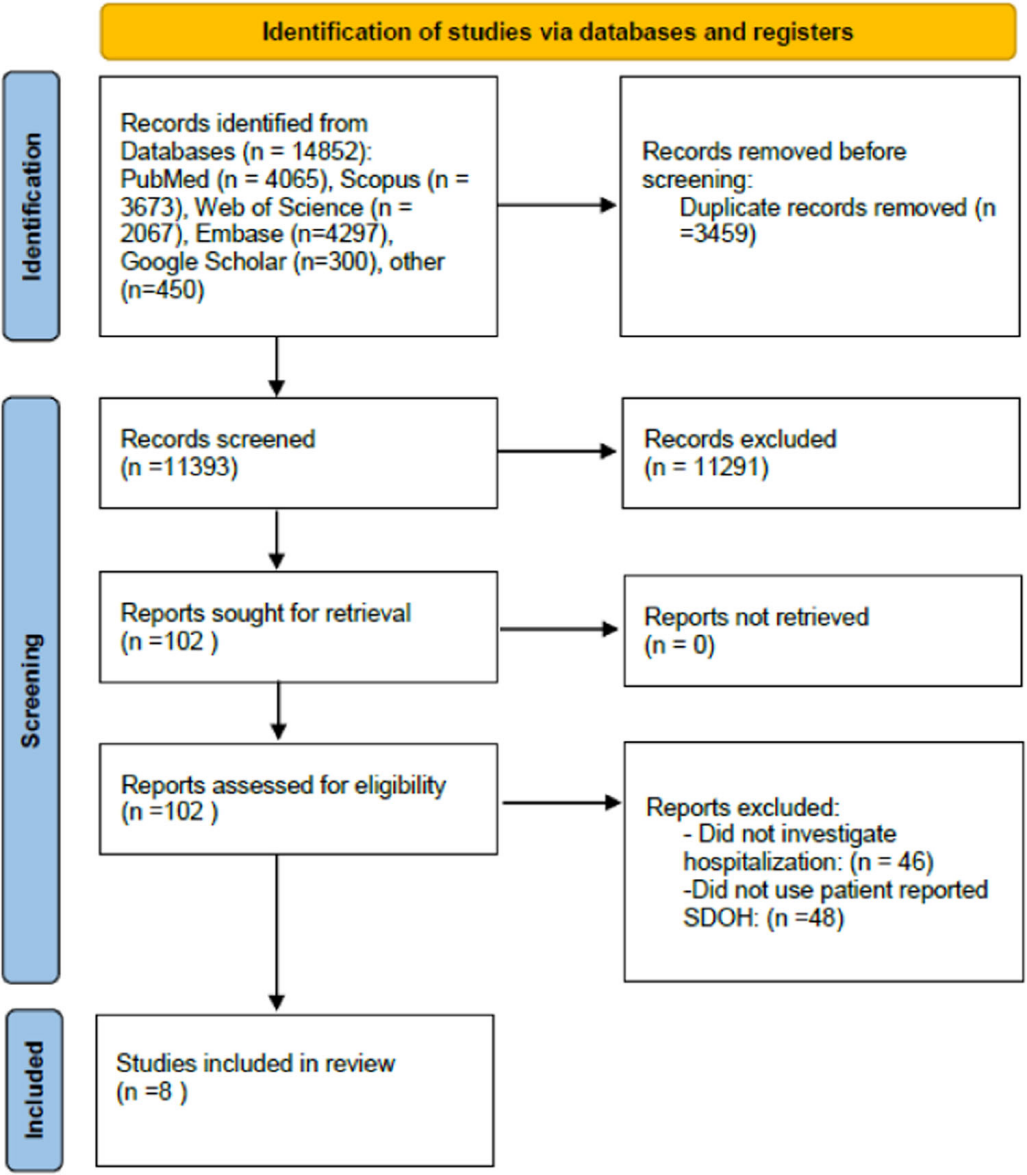
The flow diagram of the screening process. SDOH, Social Determinants of Health.

Our search yielded 14,852 citations. After duplicate removal (*n* = 3459) and screening, eight studies^8^, ^9^, ^10^, ^11^, ^12^, ^13^, ^14^, ^15^ met the criteria to be reviewed (Figure 1). All of the studies were published from 2020 to 2022. All of the studies were undertaken in the United States. The sample sizes ranged from 226^9^ to 56,155^15^ participants. Key study findings are summarized in Table 2. Two studies^10^, ^15^ investigated the impact of the cumulative number of SDOH on hospitalization, while another two^9^, ^14^ determined the effect of each SDOH domain on hospitalization rate, and one^13^ investigated both. In three studies,^8^, ^11^, ^12^ latent class analysis was applied to divide participants based on their social risks. In terms of study design, six^8^, ^10^, ^12^, ^13^, ^14^, ^15^ were cross‐sectional, and two^9^, ^11^ were retrospective cohort studies. The quality of the included studies is reported in Table S2.

**Table 2:** A summary of the key findings of the studies.

Author, year	Description of the used method (*n* = sample size)	Reported outcomes of hospitalization
Blalock et al. (2021)^8^	The authors examined veterans at high risk for hospitalization through a mail survey on SDOH. The research team used eleven self‐reported items known to impact hospital admission and to be sensitive to the intervention to classify participants based on their social risk using latent class analysis. (*n* = 4684)	Applying latent class analysis, five subgroups were identified: “minimal SDOH vulnerabilities” (8% hospitalization rate), “poor/fair health with few SDOH vulnerabilities” (12% hospitalization rate), “social isolation” (10% hospitalization rate), “multiple SDOH vulnerabilities” (12% hospitalization rate), and “multiple SDOH vulnerabilities without food or medication insecurity” (10% hospitalization rate). The “Multiple SDOH vulnerabilities” subgroup showed a higher risk of 180‐day hospitalization than those with “minimum SDOH vulnerabilities” (OR: 1.53).
Canterberry et al. (2022)^15^	A sample population of Medicare beneficiaries was surveyed for health‐related social needs. The data were linked to medical claims, and a regression model was applied to assess the association between social needs and healthcare utilization. (*n* = 56,155)	Compared to those without social needs (SN), those with any SN (OR: 1.53), one SN (OR: 1.35), two SN (OR: 1.68), three SN (OR: 1.57), four SN (OR: 1.82), and five or more SN (OR: 2.12) had higher odds of avoidable hospital stays.
Foster et al. (2020)^9^	Children (0–18 years) eligible for Supplemental Security Income and Medicaid were included in the research. Multivariable hurdle Poisson regression was used to assess the connection between SDOH and one‐year hospital and ED utilization. (*n* = 226)	The ED visit rate was 55% (mean: 1.5 per year). The incidence of hospitalization was 20% (mean: 0.4 per year). Patients with a history of “unaddressed housing insecurity” (rate ratio: 1.55) or a “safety concern” (rate ratio: 2.04). had a higher annual ED usage rate among persons who had >0 ED Visits or Hospitalizations.
Jones et al. (2022)^10^	6000 patients from seven primary care clinics were surveyed. 1748 were matched to medical claims. A two‐part model was used to assess the impact of SDOH on healthcare utilization. A modified logistic regression model was used to estimate risk ratios for cumulative SDOH variables and self‐reported chronic illnesses. (*n* = 1748)	Three or more SDOH needs were associated with an increased incidence of ED (aRR: 1.61) and inpatient (aRR: 1.76) visits.
MacCarthy et al. (2020)^11^	A retrospective analysis of Medicaid beneficiaries utilizing a combination of patient‐reported SDOH and Medicaid claims. By latent class analysis, participants were divided into four social risk classes. (*n* = 8943)	With each higher (worse) social risk class, the adjusted log relative rates of both primary care visits and visits to the ED were higher. Participants who were “unemployed and had many social risks” (the highest social risk class) had a log relative primary care treatable rate of 39% and a log relative need for ED care rate of 29%, after adjusting for age, gender, and severity of illness.
Rogers et al. (2020)^12^	Social needs were screened among a population of predicted high healthcare utilizers. Latent class analysis was applied to categorize the participants based on their reported SDOH. (*n* = 2,533)	Participants were separated into four social risk classes based on latent class analysis. Class 1 consisted of people with four or more self‐reported risks, and class 4 consisted of participants with no self‐reported risks. Despite having a lower Charlson comorbidity score, class 1 patients had considerably more total inpatient visits than class 4 patients (1.5 vs. 1.1, *p* < 0.001).
Wray et al. (2021)^13^	A cross‐sectional study assessed the association of each SDOH and cumulative SDOH burden with hospitalization using a patient‐reported SDOH survey. (*n* = 55,186)	Hospitalized participants reported greater educational deficits (67%), economic instability (33%), food insecurity (14%), lack of community (14%), less access to healthcare (6%), and more social isolation (34%) compared with nonhospitalized individuals.
Zulman et al. (2020)^14^	Veterans who had a 1‐year risk of hospitalization or death in the 75th or higher percentile were eligible to participate. The major outcomes of interest were all‐cause hospitalization 90 and 180 days after completion of the SDOH survey. (*n*=4,685)	Based on the Akaike information criterion, the regression model with survey‐based covariates and electronic health records‐based covariates predicted hospital admission at 90 days and 180 days more accurately than restricted models with only electronic health records‐based covariates.

Abbreviations: aRR, adjusted risk ratios; ED, emergency department; SDOH, social determinants of health; SN, social need.

Studies involving latent class analysis combined the patient‐reported social risks to ascertain social risk classes and categorized the participants in these classes. In the study of Blalock et al.,^8^ by applying latent class analysis, the odds ratio for 180 days of hospitalization was reported to be 1.47 (0.89–2.42) for those in the “poor/fair health with few SDOH vulnerabilities” class, 1.53 (1.09–2.14) for those in the “multiple SDOH vulnerabilities” class, 1.21 (0.78–1.87) for those in “multiple SDOH vulnerabilities without food and medication insecurity” class, and 1.18 (0.75–1.86) for those in the “social isolation” class, assuming those with “minimal SDOH vulnerabilities” as the reference group. The authors concluded that self‐reported SDOH assessments may highlight key subgroups for whom targeted interventions can be offered to lower their risk of hospitalization and other adverse outcomes. Also, McCarthy et al.^11^ used latent class analysis to categorize individuals into four groups based on their social risk. Those in the “unemployed and many social risks,” “unemployed and limited internet and car access,” and “employed and high financial strain” classes had 17% (9–25), 47% (40–54), and 80% (70–90) higher adjusted log relative rates for requiring emergency department care, respectively, than those in the lowest social risk class when adjusted for sex, age, and illness severity. Furthermore, Rogers et al.^12^ applied latent class analysis to define four classes of social risk, with class 1 possessing the “most social risk factors” and class 4 possessing the “fewest social risk factors.” The mean number of inpatient visits during the 12 month of follow‐up for class 1 (1.5 ± 2.1) was higher than for class 2 (1.3 ± 1.6), class 3 (1.1 ± 1.4), and class 4 (1.1 ± 1.5).

Also, the cumulative impact of SDOH was investigated by Jones et al.^10^ They demonstrated that the pattern of healthcare utilization is intertwined with SDOH needs. According to their results, those with 1–2 SDOH needs (OR: 1.34, 0.81–2.16) and 3 or more SDOH needs (OR: 1.76, 1–3.05) had higher odds of inpatient care utilization compared to individuals without SDOH needs. Based on these results, they suggested that SDOH needs to be considered during the healthcare process. Also, Canterberry et al.^15^ studied a sample of Medicare beneficiaries and concluded that those with a higher number of social needs are more prone to hospitalization (five or more social needs, OR: 2.12 vs. those without social needs). In the study of Wray et al.,^13^ social isolation (OR: 1.17, 1.08–1.26), lower education (OR: 1.12, 1.02–1.25), and food insecurity (OR: 1.36, 1.22–1.52) were associated with hospitalization in the adjusted analysis. In comparison with individuals who reported no SDOH needs, increased hospitalization rates were found in those with 3–4 SDOH (OR: 1.25, 1.06–1.49) or 5 SDOH (OR: 1.72, 1.40–2.06). In the study by Foster et al.,^9^ although statistically insignificant, previously unaddressed housing (OR: 2.18, 0.88–5.40) or food (OR: 1.44, 0.51–4.08) insecurity was associated with hospitalization. Finally, Zulman et al.^14^ showed that adding survey‐based covariates to electronic health records‐based covariates improves the prediction of hospital admission at 90 days and 180 days compared to models based only on data from electronic health records.

Table 3 presents the SDOH domains in each study according to the modified KFF framework. All eight studies investigated the relationship between food security and hospitalization rate. Six studies investigated economic status, and five looked at the social context. Health literacy was evaluated in two studies. Studies utilized different frameworks to define SDOH, outlined in Table S2. Data‐gathering methods differed between the studies and included mail, email, and telephone surveys^8^, ^10^, ^12^, ^14^, ^15^ or in‐person interviews.^9^, ^11^, ^13^

**Table 3:** The domains of social determinants of health (SDOH) reported in the included studies.

Study	Economic stability	Neighborhood and physical environment	Education	Food security	Community and social context	Healthcare system	Mental health	Health literacy
Blalock et al.^8^				*	*		*	
Canterberry et al.^15^	*	*		*			*	
Foster et al.^9^		*		*				
Jones et al.^10^	*		*	*				
McCarthy et al.^11^	*		*	*	*		*	
Rogers et al.^12^	*	*		*	*	*		*
Wray et al.^13^	*	*	*	*	*	*		
Zulman et al.^14^	*		*	*	*		*	*

## 4 DISCUSSION

We conducted a scoping review of the association between patient‐reported social risks and hospitalization rate. Although the methods and questionnaires used to determine the SDOH varied substantially, nearly all studies hinted at the significant impact of SDOH on the odds of hospitalization. This association was more significant when studies investigated the groups with most social risk factors.

Because of the interconnectedness of different SDOH domains, it is debatable whether treating each domain as a separate indicator or measuring the cumulative number of social risks is more advantageous. The SDOH domains are connected across a continuum. Through increased allostatic load and stress, social risks contribute to poor mental health outcomes, which in turn negatively impacts physiological systems.^10^, ^29^, ^30^ On the other hand, mental health issues are linked to increased healthcare utilization and costs.^31^ Or for instance, neighborhood safety issues and segregation may lead to decreased healthcare access and a poorer social context,^32^, ^33^ all of which contribute to higher rates of diseases and subsequent hospitalization,^34^, ^35^ as demonstrated in the included studies.^13^, ^15^ As mentioned in the results section, some studies analyzed the cumulative impact of the number of social needs, while others used latent class analysis to examine social needs as a whole. While every SDOH need is important, it may be more beneficial to examine a constellation of social needs since it paints a more holistic picture.^13^ However, there is a paucity of studies investigating SDOH as a whole; it is suggested to collect the SDOH needs of individuals before or during healthcare utilization.^36^

All of the included studies investigated the impact of food security issues on hospitalization rates. The consequences of SDOH needs on healthcare usage may vary depending on the individual's physical and mental health condition. For example, food insecurity may be more pressing for diabetic patients (who must control their blood glucose levels) than for individuals with mental health issues.^11^, ^37^ To address these discrepancies, patients' underlying illnesses should be investigated when assessing the relationship between social risks and hospitalization. Health literacy and healthcare access were measured only in two studies. Hence, future studies should focus on these under‐investigated areas of hospitalization‐related SDOH.

The complexity of SDOH renders it challenging to develop a screening tool that is both concise and comprehensive. In a recent systematic review, 21 tools were identified for social risk screening; however, they evaluated between three to six social risk domains (median: four domains), with only two tools evaluating all six domains.^38^ All tools assessed neighborhood or physical environment risks, while nine assessed healthcare access.^38^ Sarmento et al.^39^ developed a questionnaire on determinants of potentially avoidable hospitalization, mainly covering disease self‐management, social support, health literacy, health status, and lifestyle at the individual level, and healthcare access and environmental characteristics at the contextual level. Although social risk screening tools are available in the literature,^40^, ^41^ there is a paucity of appropriate and comprehensive instruments for social risks that may have a greater impact on hospitalization.

An ethical consideration arising from the patient‐reported SDOH is collecting patient information on some issues that healthcare organizations cannot address.^8^ Although healthcare organizations can work to dismantle institutional racism and provide equitable care (considering racism as an example of a social risk), most healthcare organizations are not capable of addressing housing insecurities or neighborhood problems. As a result, there should be a careful evaluation of the research projects in this regard to maintain patients' information privacy. It is also worth noting that some countries seek to address non‐healthcare‐related factors that influence health outcomes through “social prescribing”, which is described elsewhere.^42^, ^43^

There is evidence suggesting that patients with social risks do not want assistance from the healthcare system.^44^, ^45^ Some patients may want their healthcare providers to be aware of their social needs and to offer socially informed care (e.g., prescribing more affordable medication and offering telehealth visits to reduce transportation costs). Furthermore, the ethical issues of collecting data on patients' social needs are just as important to discuss. For example, having a patient fill out a written or electronic form on which they identify a social need that is not acknowledged by a member of their healthcare team may cause distress or mistrust and lead to inaccurate data. Holding a patient‐centered conversation about social risks and acknowledging patients' needs in an empathetic and thoughtful manner may help improve patient‐provider relationships.

Studies have shown that social‐need assistance programs are more effective in preventing inpatient visits than emergency department visits.^46^, ^47^ An encouragement‐designed randomized study by Brown et al.^47^ was conducted among those adult Medicaid beneficiaries at the top 15% risk for healthcare use. Participants were randomized to intervention (social needs case management for 12 months) and control groups. Participants in the intervention group had lower rates of all inpatient admissions (OR: 0.89, CI: 0.81–0.98) and avoidable inpatient admissions (OR: 0.72, CI: 0.55–0.88). However, when comparing the costs of the assistance program and savings through the decreased emergency department and inpatient visits, it was revealed that savings were not covering the costs of the program. Hence, the financial benefit of assistant programs remains under question, and further research should focus on establishing the features of a successful program. This study investigated the hospitalization cost in the short term, but the potential impact of addressing social needs on general quality of life and more long‐term health outcomes cannot be denied. Future research should investigate the cost‐effectiveness of social interventions more comprehensively.

This is the first review to our knowledge to elucidate the link between patient‐reported social risks and hospitalization. However, several limitations have to be taken into consideration. Our analysis revealed the scarcity of studies, which can serve as a call to action, especially in countries other than the United States, to provide a more comprehensive grasp of the subject. This was possibly due to merely looking at hospital utilization rates and patient‐reported SDOH. Also, we used a search strategy that included keywords related to “social determinants of health” (Table S1). But we should admit that we did not include search terms for every part of the SDOH because that would make the screening process impractical. Furthermore, most of the research examined was cross‐sectional, making it impossible to prove causation. Hence, future studies should consider prospective designs and collect longitudinal data. Also, we limited our inclusion criteria to patient‐reported SDOH. However, census‐based data or other predefined data could be used as a stand‐in for some SDOH domains. Another limitation was that the components of SDOH were not homogenously measured in different studies, that is, justifiable through the subjective nature of social studies. Furthermore, as with any review study, some studies may have been missed despite our systematic approach. Given the difficulties in collecting and validating patient‐reported SDOH data, the results of this review should be interpreted with caution.

## 5 CONCLUSION

Individuals with social risks may be more prone to hospitalization. This may lay the groundwork for developing new approaches that reduce hospitalization. There is a need for a paradigm shift to meet the patients' social risks with the short‐term purpose of improving the quality of life and the long‐term purpose of lowering hospitalization rates. Our results imply that screening for SDOH vulnerabilities in patients at high risk of hospitalization and directing those with multiple social risks to relevant medical and psychosocial services might be valuable. Furthermore, as SDOH varies substantially in different populations, more studies should be done in different parts of the world to reach a more comprehensive understanding of this topic.

## AUTHOR CONTRIBUTIONS

**Ali Ardekani**: Conceptualization; data curation; formal analysis; investigation; methodology; project administration; software; supervision; validation; visualization; writing—original draft; writing—review & editing. **Reza Fereidooni**: Conceptualization; formal analysis; methodology; validation; visualization; writing—original draft. **Seyed Taghi Heydari**: Conceptualization; formal analysis; investigation; software; writing—review & editing. **Sulmaz Ghahramani**: Formal analysis; investigation; methodology; project administration; resources; supervision; validation; writing—review & editing. **Saeed Shahabi**: Investigation; methodology; project administration; visualization; writing—review & editing. **Kamran Bagheri Lankarani**: Conceptualization; investigation; methodology; project administration; supervision; writing—review & editing.

## TRANSPARENCY STATEMENT

The lead author Ali Ardekani affirms that this manuscript is an honest, accurate, and transparent account of the study being reported; that no important aspects of the study have been omitted; and that any discrepancies from the study as planned (and, if relevant, registered) have been explained.

## Supporting information

Supporting information.

## Data Availability

All of the extracted data are represented within the manuscript.
