# Supplementary material for: The association of patient‐reported social determinants of health and hospitalization rate: A scoping review
Source: Health Sci Rep. 2023 Feb 22;6(2):e1124. doi: 10.1002/hsr2.1124 (PMC9944244; doi:10.1002/hsr2.1124)
Supplement: Supplementary file 1 — Supporting information. [file HSR2-6-e1124-s001.docx]

| Database | Search strategy |
| --- | --- |
| PubMed | ((social determinant of Health[MeSH Terms]) OR ("social determinant*"[Title/Abstract]) OR ("social need*"[Title/Abstract]) OR ("social risk*"[Title/Abstract])) AND (("Hospital*"[Title/Abstract]) OR (Hospitals[MeSH Terms]) OR (Hospitalization[MeSH Terms])) |
| Scopus | (INDEXTERMS("social determinant of Health") OR TITLE-ABS-KEY("social determinant*" OR “social need*” OR “social risk*”)) AND (INDEXTERMS(hospital) OR TITLE-ABS-KEY("hospital*")) |
| WoS | (TS=("social determinant*" OR “social need*” OR “social risk*”) ) AND (TS=(hospital*) ) |
| Embase | ('social determinants of health'/exp OR 'social determinant*':ab,ti OR 'social risk*':ab,ti OR 'social need*':ab,ti) AND ('hospital'/exp OR 'hospitalization'/exp OR 'hospital*':ab,ti) |
| Google Scholar^1^ | “social determinant*” AND “hospital*”  “social risk*” AND “hospital*”  “social need*” AND “hospital*” |
| Hand searched journals^2^ | Lancet Public Health (<https://www.thelancet.com/journals/lanpub/home>)  JMIR Public Health and Surveillance (<https://publichealth.jmir.org/>)  American Journal of Public Health ( <https://ajph.aphapublications.org/>)  Health Policy ( <https://www.sciencedirect.com/journal/health-policy>)  Social Science & Medicine (<https://www.journals.elsevier.com/social-science-and-medicine>)  International Journal of Health Policy and Management (<http://www.ijhpm.com/>)  Frontiers in Public Health ( <https://www.frontiersin.org/journals/public-health>)  BMC Health Services Research (<https://bmchealthservres.biomedcentral.com/>)  Archives of Public Health ( <https://archpublichealth.biomedcentral.com/>)  International Journal of Public Health ([www.springer.com/public%20health/journal/38](http://www.springer.com/public%20health/journal/38))  Health Services Research (<https://onlinelibrary.wiley.com/journal/14756773>)  Health Policy and Planning ( <https://academic.oup.com/heapol>)  Journal of Public Health ( <https://academic.oup.com/jpubhealth>)  Health Research Policy and Systems ( <https://health-policy-systems.biomedcentral.com/>)  Public Health ( <https://www.journals.elsevier.com/public-health>)  European Journal of Public Health ( <https://academic.oup.com/eurpub>)  BMC Public Health ( <https://bmcpublichealth.biomedcentral.com/>)  Journal of Public Health Policy ( <https://www.palgrave.com/gp/journal/41271>)  Journal of General Internal Medicine ( <https://www.springer.com/journal/11606>)  JAMA Network Open ( <https://jamanetwork.com/journals/jamanetworkopen>)  PLOS ONE ( <https://journals.plos.org/plosone/>)  BMJ Open ( <https://bmjopen.bmj.com/>) |
| ^1^The first 100 citations were screened for each strategy.  ^2^ The results of the first two pages for each journal were searched using “social determinant*” AND “hospital*”. The journals were searched from August 25^th^ to August 27^th^ . | |

**Table S1.** Search strategies.

**Table S2.** The extracted data regarding social determinants of health frameworks used in each study and methods of data gathering.

| Study | Framework |
| --- | --- |

| Blalock et al. [1] | “A mail survey was fielded in 2018 to a nationally-representative stratified random sample of 10,000 Veterans who had at least one veterans affairs (VA) outpatient visit between 3/20/2017 and 3/18/2018, and were considered “high risk” (defined as 1-year risk of hospitalization or death in the ≥ 75th percentile of VA’s Care Assessment Need (CAN) score on 3/16/2018.19 CAN score estimates probability of hospitalization or death within one year and is calculated based on demographics, medical conditions, vital signs, prior year Veteran Health Administration (VHA) health services utilization, medications dispensed, and laboratory results. Using the Dillman method [2], the survey was sent with a cover letter, $2 bill incentive, prepaid return envelope, and 1–800 telephone number or return postcard to opt-out. The cover letter described the purpose of the survey, how Veterans were identified, and a statement that return of the mailed survey constituted informed consent. Veterans who did not opt out and who did not respond within a 6-week period were mailed a second survey with a prepaid envelope and cover letter.”  “Survey measures were informed by the cycle of complexity model [3], which posits that patient complexity is a multifactorial construct comprised of workload, acute shocks and medical events, capacity/resilience, and access/utilization. The domains in this theoretic framework provided a structured way to identify a preliminary list of measures by domain instead of choosing a set of measures without an underlying logic. The preliminary list was reduced to the one-third that the study team prioritized because they were of greatest interest, brief, and (when possible) validated. While the initial paper from these data examined all 22 measures individually for prediction of 90- and 180-day hospitalization [4], the current investigation is limited to an a priori subset of 11 measures for creation of patient-level subgroups.” |
| --- | --- |
| Canterberry et al. [5] | "Adult, non-institutionalized, enrollees in an individual Medicare Advantage plan offered by Humana Inc. on October 1, 2019 were eligible for outreach. Eligible individuals were sampled at the household level. Among households with two or three eligible individuals, only one was randomly selected for participation. Outreach to eligible individuals occurred over a rolling period, with a maximum of eight contact attempts per individual, and was accomplished using interactive voice response (IVR) phone call, text messaging and email, based on the availability of valid contact information within each channel. Survey completion by channel was as follows: IVR (66%), Text (30%), and email (4%). The survey was administered in English and Spanish. Multi-answer questions were converted to distinct questions for better clarity over IVR."  “The authors used a survey “adapted from the Center for Medicare and Medicaid Services (CMS) Accountable Health Communities (AHC) HRSNs Screening Tool.” [6] The survey assessed the presence of 7 HRSNs: (1) food insecurity, (2) financial strain, (3) loneliness, (4) unreliable transportation, (5) utility insecurity, (6) housing insecurity, and (7) poor housing quality.” |
| Foster et al. [7] | “Enrolled patients and families were assigned a paired registered nurse case manager and nonclinical care coordinator, who had undergone program-specific training. Case managers had backgrounds in primary care, mental health, and/or public health, and coordinators had backgrounds in parenting support, health education, school health, and/or community resource navigation. Three coordinators were certified in Spanish proficiency. Each complementary case manager and coordinator pair was assigned on the basis of family-identified need. For example, a family needing help with a school-individualized education plan was assigned to a coordinator with school-based experience, whereas another family with low health literacy was paired with a coordinator who had a background in health education.”  “A case manager completed the intake assessment during patient enrollment. The intake assessment was a non-validated survey that included open-ended questions about communication preferences and SDOH thought to impact the patient’s care or family functioning; for consistency, coordinators were trained in the use of standardized prompts. If identified in the assessment, food insecurity was addressed by providing families with information and application assistance with programs, such as the Women, Infant, and Children Program or Supplemental Nutrition Assessment Program. Housing insecurity also was addressed by providing help applying for housing assistance programs. CM for caregiver health or a safety concern was tailored on the basis of the specific elicited concern. For example, family caregivers who indicated emotional strain would receive mental health information. A caregiver with intimate partner violence was referred to safe harbor resources.” |
| Jones et al. [8] | “Participants were selected from electronic health records between August 2018 and February 2019 in 4 waves of 1500 for a total population of 6000. Patients were required to be at least 18 years old and have had a recent (within a 30-day window) visit to 1 of the 7 study clinics. Each patient was assigned a unique identifier, mailed a paper survey with a $5 cash incentive, and simultaneously sent an e-mail to complete an online survey option. A reminder letter, e-mail, and phone call follow-up was conducted for non-respondents. In total, 2380 individuals completed the survey (39.7% response rate).”  “Insurance identifiers from survey respondents’ electronic health records were sent to the health plan of the health care system and matched, where possible, to the health claims data. Claims data were requested for the period from January 2018 to December 2019, so respondents could have at least 6 months of health care utilization either side of their qualifying clinic visit. In total, 1899 individuals (79.8% of respondents) were matched to health claims data. Respondents with <6 months of claims eligibility before their visit were excluded from analysis giving a final claims sample of 1748 (73.4% of respondents).”  “The survey instrument was designed in partnership with the community health division of the nonprofit health care system. The survey included self-reported items on chronic physical and behavioral health conditions, a checklist of SDOH and medical-related need (based on services offered by the clinic-based community resource desk), and a set of demographic questions (Supplementary Appendix SA). The predictor for the study was constructed from a list of 8 SDOH needs. Participants were asked if they had recently needed assistance with food, utility costs, transportation, clothing, housing/rent, services for children, jobs or employment, or education classes. Responses were summed (1 = needed assistance, 0 = no need) and categorized into ‘‘No SDOH need,’’ ‘‘1–2 SDOH needs,’’ and ‘‘3 or more SDOH needs.’’ |
| McCarthy et al. [9] | “To be eligible for the study, patients had to be aged between 18 and 64 years, be insured by the District of Columbia Medicaid program, have access to a telephone, and be present at 1 of the aforementioned acute or ambulatory care sites affiliated with the 2 facilities between September 2017 and December 2018. Patients were excluded if they were unable to understand consent, non-English speaking, too sick (ie, triage acuity level 1), or also insured by Medicare. Patients willing to participate signed a written consent form and agreed to complete an interview during their medical encounter, allowed a research assistant to abstract relevant study data from their medical record, and permitted the study team to obtain a copy of their Medicaid claims. During the 16-month enrollment period, we screened 17,719 patients; 12,346 were eligible. The most common reason for ineligibility (85%) was that the patient had already been enrolled. Among the 3,403 eligible patients who were not enrolled, 55% refused, 24% reported feeling too uncomfortable, and 21%completed their medical visit before the research assistant was able to approach them, resulting in a final sample of 8,943 participants. Because enrollment occurred at 2 EDs with much higher patient volumes and longer hours of operation than either of the clinics, the majority of study enrollment (87%) occurred at the ED visit.”  “The social determinants of health survey was developed in accordance with the World Health Organization social determinants of health conceptual model and included measures of structural and intermediary determinants of health.12 The model posits that structural determinants of health such as education, income, and occupation define a person’s socioeconomic position in society. In turn, socioeconomic status shapes intermediary social determinants, including material circumstances, health behaviors, and psychological factors. Structural and intermediary social determinants of health affect the incidence of illness and injury, as well as the ability of people to manage their health problems. Our survey included 35 questions and took approximately 10 minutes to complete and consisted largely of questions or short scales previously validated by others [10]. The survey included the following structural determinants of health: sexual orientation, highest level of education achieved, employment status, and employment duration. The intermediary determinants we asked participants about included measures of material circumstances (ie, food insecurity and financial strain), health behavior questions (ie, smoking and alcohol use), and psychological factors (ie, marital status and loneliness).” |
| Rogers et al. [11] | “We utilized prospectively collected social risk data gathered from a social needs intervention for adult predicted healthcare high utilizers and applied latent class analysis (LCA) based on 14 patient-reported social risks: food insecurity, healthy food, housing, housing safety, employment, transportation, financial security, utility assistance, public benefits, financial counseling, affordable medical care, health literacy, caregiver support, and social support.”[12] |
| Wray et al. [13] | “We used cross-sectional data from the 2016–2018 National Health Interview Surveys (NHIS), a national sample of noninstitutionalized individuals residing within the US, conducted annually by the National Center for Health Statistics at the Centers for Disease Control and Prevention.13 The NHIS uses computer-assisted personal interviewing to annually administer the survey and collect health-related information from respondents. During the assessed years, the unconditional final sample adult response rate ranged from 53.0 to 54.3%. This study used publicly available data and was exempt from institutional review board review.”  “After limiting the sample to adults ≥18 years and excluding individuals with missing data on hospitalization (<1%), our analytic sample included 55,186 respondents—representing more than 246 million Americans. To assess whether someone had been hospitalized in the previous year, we used the question: “Have you been hospitalized overnight in the past 12 months? Do not include an overnight stay in the emergency room.””  “We adopted and modified the Kaiser Family Foundation (KFF) model on Social Determinants of Health to classify specific NHIS questions into pre-defined domains of social risk. Briefly, the KFF model consists of six domains (economic stability, neighborhood and physical environment, education, food insecurity, community and social context, and health care access) that describe social elements that may adversely impact an individual’s health.14 The NHIS questionnaires were assessed for questions that addressed each of the six domains. All questions were discussed among the authors and categorized into the most appropriate domain (Table 2). To maximize the sensitivity of our assessment, respondents were considered to have a SDOH if they answered positively to any question within each of the domains.” |
| Zulman et al. [4] | “The sampling frame included veterans with a 1-year risk of hospitalization or death on March 16, 2018, that was in the 75th or higher percentile based on the VA’s Care Assessment Need score,36,37 the lower end of which reflects approximately 10% to 11% probability of hospitalization or death in the upcoming year. The 1-year risk measure is more stable and should fluctuate over time more slowly than a 90-day risk measure. A sample of 10 000 high-risk veterans within US VA medical centers was selected, using PROC SURVEYSELECT (SAS, version 9.4; SAS Institute Inc), via stratified random sampling proportional to the percentage of veterans with Care Assessment Need score 75 or higher in each US VA medical center to obtain a nationally representative sample of veterans who had at least 1 VA outpatient visit from March 20, 2017, to March 18, 2018, were alive, and had a valid home address at the time of cohort generation”  “Survey measures were informed by the Cycle of Complexity model, which posits that patient complexity is a multifactorial construct composed of workload, acute shocks and medical events, and capacity or resilience, all of which influence access or use. Study team members used an iterative consensus process to generate a draft list of survey measures, prioritizing measures that were not available in administrative data, had a known association with hospitalization, explained the maximum amount of variation within the construct, shared minimal overlap with other scales, and could help primary care teams identify patients who would benefit from clinical intervention.” |

**References**:

1. Blalock, D.V., et al., *Subgroups of High-Risk Veterans Affairs Patients Based on Social Determinants of Health Predict Risk of Future Hospitalization.* Med Care, 2021. **59**(5): p. 410-417.

2. Dillman, D.A., *Mail and telephone surveys: The total design method*. Vol. 19. 1978: Wiley New York.

3. Zullig, L.L., et al., *A Systematic Review of Conceptual Frameworks of Medical Complexity and New Model Development.* J Gen Intern Med, 2016. **31**(3): p. 329-37.

4. Zulman, D.M., et al., *Patient-Reported Social and Behavioral Determinants of Health and Estimated Risk of Hospitalization in High-Risk Veterans Affairs Patients.* JAMA Netw Open, 2020. **3**(10): p. e2021457.

5. Canterberry, M., et al., *Association Between Self-reported Health-Related Social Needs and Acute Care Utilization Among Older Adults Enrolled in Medicare Advantage.* JAMA Health Forum, 2022. **3**(7): p. e221874.

6. Alley, D.E., et al., *Accountable health communities—addressing social needs through Medicare and Medicaid.* N Engl J Med, 2016. **374**(1): p. 8-11.

7. Foster, C.C., et al., *Social Determinants of Health and Emergency and Hospital Use by Children With Chronic Disease.* Hosp Pediatr, 2020. **10**(6): p. 471-480.

8. Jones, K.G., S.E. Roth, and K.B. Vartanian, *Health and Health Care Use Strongly Associated with Cumulative Burden of Social Determinants of Health.* Popul Health Manag, 2022. **25**(2): p. 218-226.

9. McCarthy, M.L., et al., *The Influence of Social Determinants of Health on Emergency Departments Visits in a Medicaid Sample.* Annals of Emergency Medicine, 2021. **77**(5): p. 511-522.

10. McCarthy, M.L., et al., *Latent Class Analysis to Represent Social Determinant of Health Risk Groups in the Medicaid Cohort of the District of Columbia.* Med Care, 2021. **59**(3): p. 251-258.

11. Rogers, A., et al., *Understanding High-Utilizing Patients Based on Social Risk Profiles: a Latent Class Analysis Within an Integrated Health System.* J Gen Intern Med, 2020. **35**(7): p. 2214-2216.

12. Schickedanz, A., et al., *Impact of Social Needs Navigation on Utilization Among High Utilizers in a Large Integrated Health System: a Quasi-experimental Study.* Journal of General Internal Medicine, 2019. **34**(11): p. 2382-2389.

13. Wray, C.M., et al., *Association of Social Determinants of Health and Their Cumulative Impact on Hospitalization Among a National Sample of Community-Dwelling US Adults.* J Gen Intern Med, 2021.
